# Preoperative prediction of tumor deposits in advanced gastric cancer using intratumoral and peritumoral CT radiomics: development and validation of an ensemble model

**DOI:** 10.3389/fonc.2026.1763646

**Published:** 2026-03-04

**Authors:** Yang Yao, Pengchao Zhan, Mengchen Yuan, Yusong Chen, Yunlong Fan, Jianbo Gao

**Affiliations:** 1 Department of Gastrointestinal Surgery, The First Affiliated Hospital of Zhengzhou University, Zhengzhou, China; 2 Department of Radiology, The Third People’s Hospital of Henan Province, Zhengzhou, China; 3 Henan Key Laboratory of Imaging Diagnosis and Treatment for Digestive System Tumor, Zhengzhou, China

**Keywords:** CT, gastric cancer, preoperative prediction, radiomics, tumor deposits

## Abstract

**Objectives:**

To investigate the potential of intratumoral and peritumoral radiomics derived from CT to preoperatively predict tumor deposits (TDs) in patients with advanced gastric cancer (AGC).

**Methods:**

In this retrospective investigation, a total of 374 patients from two medical centers were recruited and divided into training (n = 186), validation (n = 80), and test (n = 108) cohorts. Intratumoral and peritumoral radiomics models were developed utilizing radiomics features derived from the corresponding 3D regions of interest (ROIs). A combined radiomics model integrating intratumoral and peritumoral features was further constructed through feature-level concatenation. Additionally, an ensemble model was established via the integration of this combined radiomics model with selected independent clinical prognostic factors. All models were evaluated using the area under the receiver operating characteristic curve (AUC), calibration curves, and decision curve analysis (DCA). Finally, the Shapley Additive Explanations (SHAP) method and nomogram were employed to elucidate the predictive mechanisms of the three radiomics models (intratumoral, peritumoral, and combined) and the ensemble model.

**Results:**

The combined intratumoral-peritumoral radiomics model showed higher AUC than the standalone intratumoral and peritumoral models across all cohorts (training: 0.874 vs. 0.751 vs. 0.830; validation: 0.846 vs. 0.720 vs. 0.713; test: 0.842 vs. 0.701 vs. 0.675). Moreover, the ensemble model yielded the highest AUCs across all cohorts (0.925, 0.865, 0.878 for training, validation, and test cohorts, respectively).

**Conclusion:**

Both intratumoral and peritumoral radiomics offer meaningful information regarding TDs, while the CT-based ensemble model holds the capacity to preoperatively predict TDs in AGC patients.

## Introduction

Gastric cancer (GC) remains a significant public health challenge globally. It is the fifth most common malignancy and the fourth leading cause of cancer-related death worldwide (1). We usually choose the appropriate treatment and assess prognosis based on TNM staging system. The TNM staging system, established by the American Joint Committee on Cancer (AJCC) and the International Union Against Cancer (UICC), is the most widely used tool for GC staging. However, an increasing number of studies have revealed the limitations of the TNM staging system (2–4). Patients with the same stage receiving identical treatments show significant variations in prognosis. Recurrence occurs even in patients who undergo radical resection for gastric cancer without lymph node metastasis. This indicates that in addition to tumor depth, gastric regional lymph node involvement, and distant metastasis, other factors also influence treatment selection and prognosis, such as tumor differentiation, lymphovascular invasion (LVI), perineural invasion (PNI), HER2 expression, MSI status, and tumor deposits (TDs) (5–8).

As a typical histopathological feature of colorectal tumors, TDs were first identified by Gabriel W.B. in 1935 (9). With the advancement in TDs research, their clinical definition has been increasingly standardized. The AJCC 8th edition TNM staging system defines colorectal cancer TDs as discrete tumor nodules within the primary tumor’s lymphatic drainage area that lack identifiable lymph node tissue, vascular structures, or neural architecture (10). Their presence indicates a higher risk of tumor dissemination and recurrence, leading to a poorer prognosis (11–13). Studies have shown that GC patients with TDs have a significantly lower 5-year survival rate than those without TDs (5, 14). However, TDs’ diagnosis mainly depends on postoperative pathology. Currently, there’s a lack of preoperative and noninvasive diagnostic tools for TDs. Preoperative diagnosis of TDs may assist clinicians in refining preoperative staging and devising perioperative treatment plans, with the potential to enhance patients’ prognoses.

Preoperative prediction of tumor deposits (TDs) in gastric cancer remains a significant clinical challenge. Previous studies have focused on assessment of TDs status using clinical characteristics: Anup et al. (12)indicated that TDs are more prevalent in Borrmann IV gastric cancer and are positively associated with deeper tumor invasion and an increased likelihood of lymph node metastasis. Li et al. (15)further reported that TDs are closely correlated with tumor diameter, Borrmann classification, tumor differentiation, pathological T stage (pT), pathological N stage (pN), pathological TNM stage (pTNM), perineural invasion, and vascular invasion. However, these studies rely on postoperative pathological features for the prediction of TDs, with limited practical utility for preoperative accurate prediction of TDs.

Notably, TDs are typically small in volume, and conventional computed tomography (CT) or magnetic resonance imaging (MRI) is constrained by scan slice thickness and spatial resolution, often failing to delineate the morphological characteristics of TDs adequately. Radiomics, a core technology in precision medicine, involves extracting high-throughput quantitative features from medical images and integrating them with machine learning algorithms to facilitate disease diagnosis, staging, treatment response prediction, and prognosis assessment (16, 17). Its application in gastric cancer has emerged as a research hotspot in recent years (18, 19).

Most prior radiomics studies have focused on intratumoral features derived from the tumor parenchyma. In contrast, peritumoral radiomics targets high-throughput quantitative characteristics of peritumoral tissues (including adjacent parenchyma, stroma, blood vessels, and fat spaces) (20). These features indirectly reflect the biological properties of the tumor microenvironment (TME), such as inflammatory responses, angiogenesis, fibrosis, and invasive potential, thereby better capturing the tumor-host interaction (21). As such, peritumoral radiomics serves as a key “imaging surrogate marker” for evaluating tumor invasiveness, metastatic potential, prognosis, and treatment response (22–24). However, the utility of both intratumoral and peritumoral radiomic features derived from CT in predicting TDs in patients with gastric cancer remains incompletely elucidated.

Therefore, the present study aimed to investigate the predictive value of CT-based intratumoral and peritumoral radiomics for TDs in AGC patients and to develop robust, interpretable models with potential clinical applicability.

## Materials and methods

### Patients and clinical characteristics

**Figure 1:**
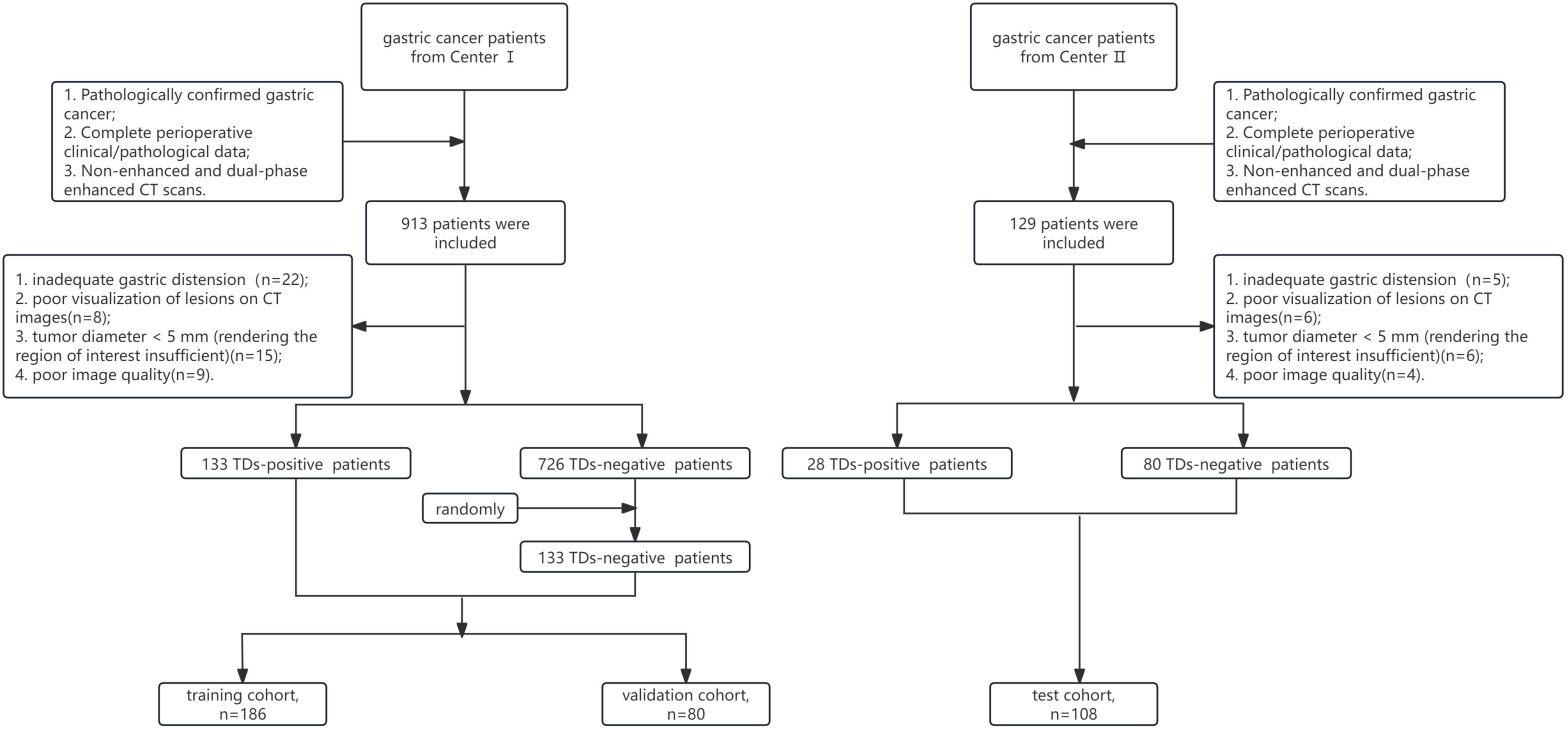
The flowchart of patients’ enrollment. TDs, tumor deposits.

The study protocol was reviewed and approved by the Institutional Review Board of our hospital. Written informed consent was waived, given the retrospective observational design of the research. Pathologically confirmed gastric cancer patients were consecutively enrolled from two medical centers. Inclusion criteria included (1): pathologically confirmed advanced gastric cancer (AGC) and undergoing gastrectomy combined with lymph node dissection (2); complete clinical and postoperative pathological data (3); availability of non-enhanced and dual-phase enhanced computed tomography (CT) scans performed within 1 week prior to treatment. Exclusion criteria included (1): inadequate gastric distension (2); poor visualization of lesions on CT images (3); tumor diameter < 5 mm (rendering the region of interest insufficient) (4); poor image quality. Between December 2015 and March 2020, a total of 913 patients were enrolled from Center I, of whom 859 were eligible. This eligible cohort consisted of 133 TDs-positive and 726 TDs-negative patients. To mitigate potential data imbalance, 133 patients were randomly selected from the TDs-negative group and matched 1:1 with the TDs-positive group. In addition, in accordance with the inclusion and exclusion criteria, a total of 108 consecutive patients were enrolled from Center II between March 2019 and March 2020 as the test cohort, of whom 28 were TDs-positive. Patients from Center I were further randomly divided into a training cohort(n=186) and a validation cohort(n=80) at a ratio of 7:3. The detailed patient inclusion and exclusion process is illustrated in **Figure 1**.

Clinical data collected in the present study comprised sex, age, tumor location, tumor thickness, maximum tumor diameter, carcinoembryonic antigen (CEA), carbohydrate antigen 12-5 (CA12-5), carbohydrate antigen 19-9 (CA19-9), and carbohydrate antigen 72-4 (CA72-4). Tumor location was categorized as cardia, body, antrum, or involvement of ≥2/3 of the gastric wall. Tumor thickness was defined as the maximal perpendicular distance from the lesion surface to the deepest infiltration point, measured in the optimal plane (axial, sagittal, or coronal). Maximum tumor diameter was additionally measured at the lesion’s maximal cross-section. CEA, CA12-5, CA19-9, and CA72–4 were dichotomized into two categories (normal vs. abnormal) based on respective reference values (CEA ≤ 3.4 μg/L, CA12-5 ≤ 35 μg/L, CA19-9 ≤ 27 μg/L, CA72-4 ≤ 6.7 μg/L).

### CT image acquisition

**Figure 2:**
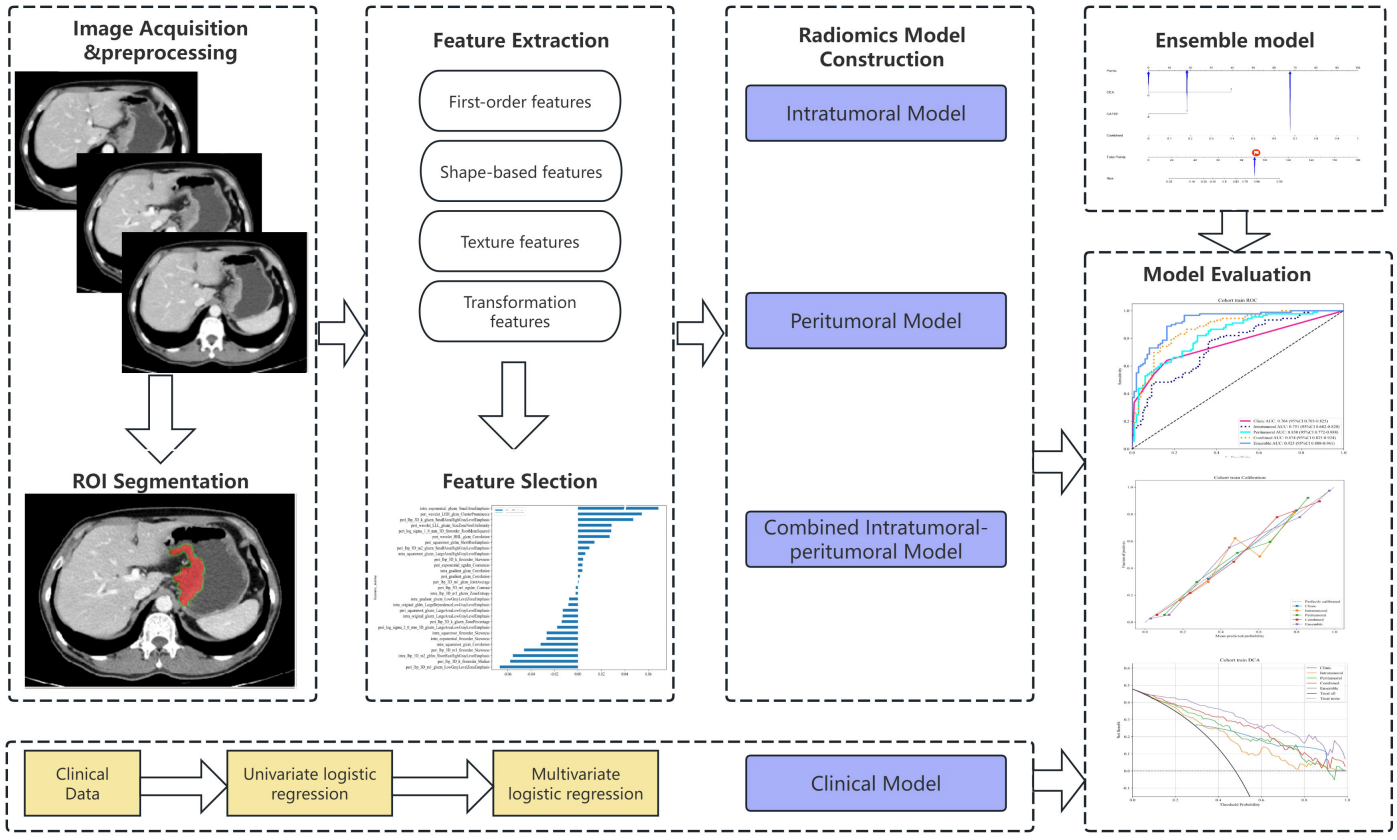
The schematic workflow of this study. ROI, region of interest.

The workflow of this study is illustrated in **Figure 2**. All patients underwent scanning using a 64-slice spiral CT scanner (Discovery CT 750 HD; GE Healthcare, Waukesha, WI, USA; or Siemens Sensation 64 CT; Siemens Healthcare, Forchheim, Germany). Patients were required to fast for at least 6 hours before scanning and received oral administration of 500–1000 ml of water to distend the gastric cavity prior to the examination. All patients were placed in the supine position, and the scanning range for both plain and contrast-enhanced CT scans covered from the top of the diaphragm to the upper edge of the pubic symphysis. Plain CT scanning was performed first. For contrast-enhanced scanning, a high-pressure injector (Urich REF XD 2060-Touch, Ulrich Medical) was used to inject the contrast agent iohexol (Shanghai Bracco Sine Pharmaceutical Co., Ltd., iodine concentration 370 mg/mL) via the antecubital vein at an injection flow rate of 3.5 mL/s and a dosage of 1.5 mL/kg. Arterial phase scanning was initiated 25–30 seconds after contrast agent injection, and venous phase scanning was started at 60–70 seconds. The scanning parameters were as follows: tube voltage of 120 kV, rotation time of 0.5−0.6 s, tube current of 250−640 mA, reconstruction section thickness of 1.25 mm and 5 mm.

### Image preprocessing and segmentation

Venous-phase images were extracted from the picture archiving and communication system (PACS) in Digital Imaging and Communications in Medicine (DICOM) format. To mitigate variability introduced by differences in scanning protocols or equipment on quantitative radiomics features, images were resampled to a voxel size of 1×1×1 mm³. Intratumoral three-dimensional (3D) regions of interest (ROIs) for gastric cancer were manually delineated slice-by-slice on axial images along the tumor boundary by YY using ITK-SNAP software (version 3.8.0; http://www.itksnap.org), with the delineation process supervised by a radiologist with 5 years of clinical experience. Following completion of intratumoral ROI outlining, a 2-mm peripheral ring was automatically generated using the SimpleITK package in Python software (version 3.6). Notably, regions outside the peritumoral area were manually excluded. To ensure intra-observer reliability, the ROIs of 30 randomly selected patients were re-delineated by YY one month later. For inter-observer reliability assessment, ZP independently performed ROI segmentation and feature extraction on the medical images of the same 30 patients. The intra-observer and inter-observer correlation coefficients (ICCs) were used to evaluate the consistency of radiomics features, with ICCs value > 0.80 indicating high consistency.

### Radiomics feature extraction

Following delineation of all ROIs, the PyRadiomics package (Version 3.6) was employed to extract intratumoral, peritumoral, and combined intratumoral-peritumoral radiomics features. Specifically, image preprocessing and transformation approaches included the original image (without additional processing), wavelet transform, Laplacian of Gaussian (LoG) transform, 2D local binary pattern (LBP2D), 3D local binary pattern (LBP3D), square transformation, square root transformation, logarithmic transformation, exponential transformation, and gradient transformation. Radiomics features encompassed first-order features, shape-based features, gray-level co-occurrence matrix (GLCM), gray-level size zone matrix (GLSZM), gray-level run length matrix (GLRLM), gray-level dependence matrix (GLDM), and neighborhood gray-tone difference matrix (NGTDM). All selected features are well-established classic radiomics descriptors.

### Feature selection criteria and dimension reduction

First, the consistency of the extracted radiomics features was assessed, and only those with ICCs ≥ 0.80 were retained. All eligible features were then normalized via Z-score normalization. A three-step sequential procedure was adopted for optimal feature selection: initially, the Mann–Whitney U test was employed to screen features with a P-value < 0.05; next, Spearman’s rank correlation analysis was performed to exclude features with a correlation coefficient > 0.9; subsequently, tenfold cross-validation was combined with the least absolute shrinkage and selection operator (LASSO) regression to identify optimal features with non-zero coefficients, which was determined by the optimal penalty parameter (λ).

### Clinical, radiomics and ensemble model development

Univariable logistic regression analysis was conducted for clinical characteristics in the training cohort, including sex, age, tumor location, tumor thickness, maximum tumor diameter, CEA, CA12-5, CA19-9, and CA72-4. Statistically significant clinical characteristics (P-value < 0.05) were subsequently included in a multivariable logistic regression model to identify independent clinical predictors, which were used for the construction of the clinical model.

Using the selected radiomic features, logistic regression was used to construct intratumoral, peritumoral, and combined intratumoral-peritumoral models. The predicted probabilities derived from the combined intratumoral-peritumoral model, along with the previously identified independent clinical predictors, were integrated into a multivariable logistic regression model to establish the ensemble model. This ensemble model was visualized using a nomogram.

### Statistical analysis

Statistical analyses and model construction/evaluation were performed using Python (version 3.6). Continuous variables with a normal distribution were presented as mean ± standard deviation (SD), while those with a non-normal distribution were expressed as median (interquartile range). For continuous variables, Student’s t-test or Mann–Whitney U test was employed based on their distribution characteristics. For categorical variables, the chi-square test or Fisher’s exact test was used, as appropriate.

The predictive performance of the models was evaluated using the area under the curve (AUC), accuracy, sensitivity, specificity, positive predictive value (PPV), and negative predictive value (NPV). Calibration curves were constructed to visually compare predicted probabilities with observed outcomes. Decision curve analysis (DCA) was conducted to quantify the clinical net benefit of each model. Differences in AUCs between models were compared using the DeLong test. The SHapley Additive exPlanations (SHAP) method was applied to interpret the radiomics models by quantifying the contribution of each feature to the model’s predictions. A two-tailed P < 0.05 was considered statistically significant.

## Results

### Clinical characteristics

A total of 374 patients with AGC were enrolled in the present study, including 161 TDs-positive patients and 213 TDs-negative patients. All patients were further divided into a training cohort (n=186), a validation cohort (n=80), and a test cohort (n=108). Detailed demographic and clinical characteristics of the patients are summarized in **Table 1**.

**Table 1:** Baseline demographic and clinical characteristics of patients.

Characteristics	Total, (n=374)	Training, (n=186)	Validation, (n = 80)	Test, (n = 108)	*p*
Age(years)	60.38 ± 10.45	61.42 ± 10.16	59.58 ± 10.56	59.18 ± 10.78	0.154
Longitude(mm)	64.35 ± 23.94	63.74 ± 20.37	62.73 ± 23.99	66.58 ± 29.12	0.492
Thickness(mm)	20.94 ± 8.93	21.24 ± 8.91	20.98 ± 9.50	20.40 ± 8.59	0.741
Sex					0.523
Male	286(76.47)	140(75.27)	65(81.25)	81(75.00)	
Female	88(23.53)	46(24.73)	15(18.75)	27(25.00)	
CEA					0.071
Normal	270(72.19)	128(68.82)	55(68.75)	87(80.56)	
Abnormal	104(27.81)	58(31.18)	25(31.25)	21(19.44)	
CA19-9					0.131
Normal	294(78.61)	140(75.27)	62(77.50)	92(85.19)	
Abnormal	80(21.39)	46(24.73)	18(22.50)	16(14.81)	
CA72-4					0.054
Normal	272(72.73)	135(72.58)	51(63.75)	86(79.63)	
Abnormal	102(27.27)	51(27.42)	29(36.25)	22(20.37)	
CA12-5					0.867
Normal	333(89.04)	164(88.17)	72(90.00)	97(89.81)	
Abnormal	41(10.96)	22(11.83)	8(10.00)	11(10.19)	
Location					0.239
Cardia	186(49.73)	91(48.92)	47(58.75)	48(44.44)	
Body	33(8.82)	14(7.53)	7(8.75)	12(11.11)	
Antrum	131(35.03)	69(37.10)	19(23.75)	43(39.81)	
Whole	24(6.42)	12(6.45)	7(8.75)	5(4.63)	

*CEA*, carcinoembryonic antigen; *CA19–9*, carbohydrate antigen 19-9; *CA72–4*, carbohydrate antigen 72-4; *CA12–5*, carbohydrate antigen 12-5.

### Clinical model

In the training set, univariate logistic regression analysis revealed that CEA, CA12-5, CA19-9, and CA72–4 exhibited statistically significant disparities between patients with positive TDs and those with negative TDs. Subsequently, multivariate logistic regression analysis identified CEA and CA19–9 as independent predictive factors for TDs status (**Table 2**). Clinical prediction models were further constructed based on these two serological biomarkers (CEA and CA19-9).

**Table 2:** Univariable and multivariable analyses of the clinical characteristics in the training cohort (n=186).

Variable	Univariable analysis		Multivariable analysis	
	Log (OR)	*p*	Log (OR)	*p*
Gender	0.928(0.773,1.115)	0.505		
Location	0.955(0.859,1.063)	0.478		
Thickness	0.997(0.987,1.008)	0.682		
Age	0.999(0.995,1.003)	0.59		
Longitude	0.999(0.995,1.002)	0.608		
CA12-5	2.667(1.213,5.859)	0.04	1.206(0.501,2.904)	0.726
CA72-4	2.923(1.723,4.958)	0.001	1.124(0.577,2.190)	0.773
CEA	4.800(2.710,8.499)	<0.001	2.770(1.358,5.652)	0.019
CA19-9	5.572(2.835,10.946)	<0.001	2.772(1.264,6.08)	0.033

*CEA*, carcinoembryonic antigen; *CA19–9*, carbohydrate antigen 19-9; *CA72–4*, carbohydrate antigen 72-4; *CA12–5*, carbohydrate antigen 12-5.

### Feature selection and model construction

A total of 1,834 radiomic features were extracted from intratumoral and peritumoral regions based on both original and processed images. For the combined intratumoral-peritumoral analysis, 3,668 radiomic features were extracted. Following consistency assessment using the ICCs, 1,178, 1,356, and 2,309 features were retained for the intratumoral, peritumoral, and combined intratumoral-peritumoral models, respectively (ICCs ≥ 0.80). Subsequent to the three-step feature selection process, 12, 16, and 27 optimal radiomic features were identified for inclusion in the intratumoral, peritumoral, and combined intratumoral-peritumoral models, respectively. Detailed information regarding these selected radiomic features is provided in **Supplementary Table 1**. These final feature sets were then employed to construct the respective intratumoral, peritumoral, and combined intratumoral-peritumoral models using logistic regression. Finally, the ensemble model was established by fusing the predictive outputs of the combined intratumoral-peritumoral model with the previously identified independent clinical predictors.

### Evaluation of model performance

**Figure 3:**
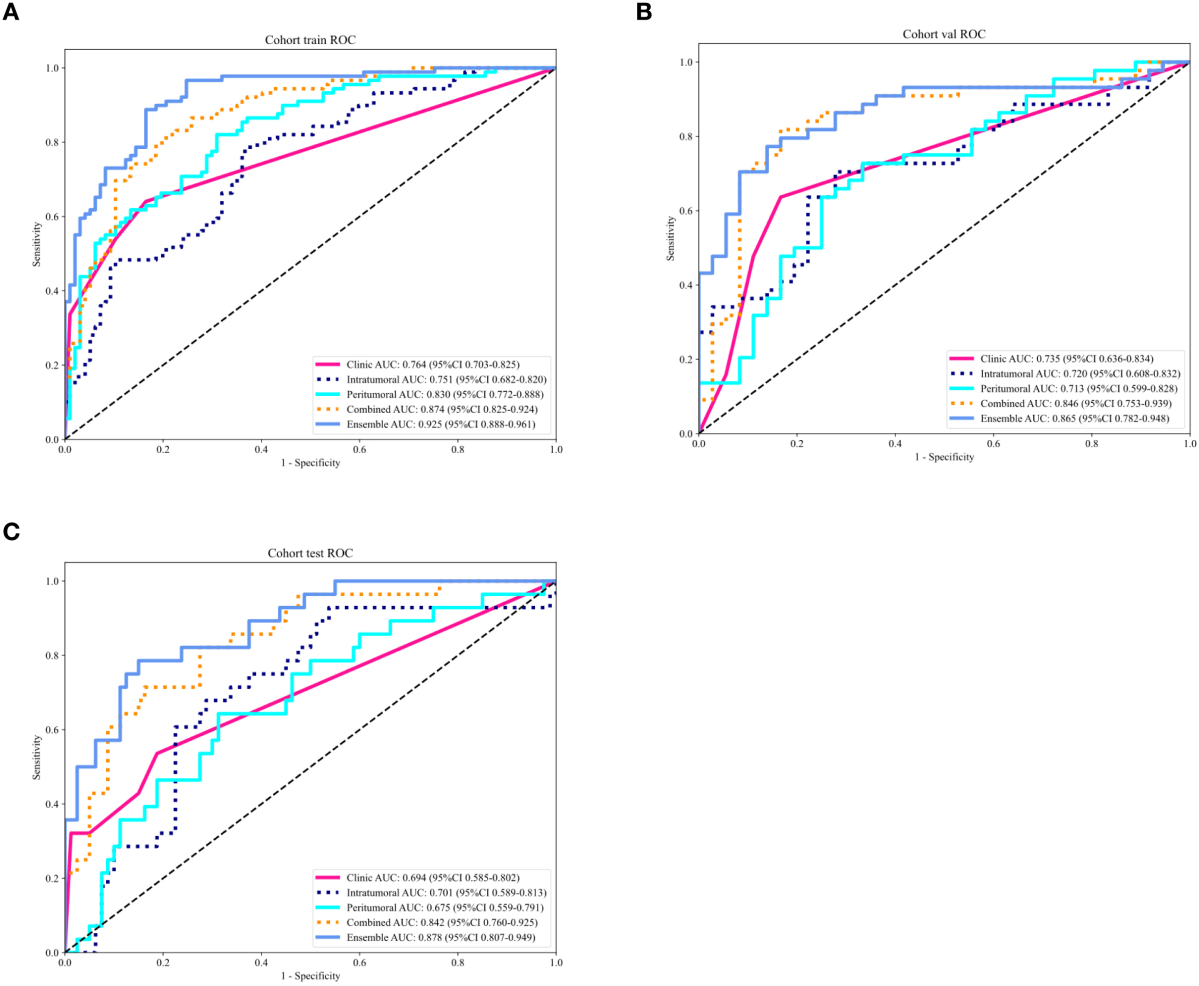
Receiver operating characteristic (ROC) curves of different models for predicting TDs status in advanced gastric cancer in the training **(A)**, validation **(B)**, and test **(C)** cohorts. AUC, area under the curve.

A total of five models were constructed in this study. The area under the curve (AUC), accuracy, sensitivity, specificity, positive predictive value (PPV), and negative predictive value (NPV) of each model for predicting TDs status in AGC are presented in **Table 3**. Receiver operating characteristic (ROC) curves for all models are illustrated in **Figure 3**. Pairwise comparisons of model performance using the DeLong test are summarized in **Table 4**.

**Table 3:** Predictive efficacy of different models for TDs status in advanced gastric cancer across the training, validation, and test cohorts.

Cohort	Models	Accuracy	Sensitivity	Specificity	PPV	NPV	AUC (95% CI)
Training	Clinic	0.742	0.640	0.835	0.781	0.717	0.764(0.703,0.825)
	Intratumoral	0.704	0.787	0.629	0.66	0.762	0.751(0.682,0.820)
	Peritumoral	0.753	0.820	0.691	0.709	0.807	0.830(0.772,0.889)
	Combined	0.806	0.820	0.794	0.785	0.828	0.874(0.825,0.924)
	Ensemble	0.86	0.888	0.835	0.832	0.89	0.925(0.888,0.961)
Validation	Clinic	0.725	0.636	0.833	0.824	0.652	0.735(0.636,0.834)
	Intratumoral	0.713	0.705	0.722	0.756	0.667	0.720(0.599,0.828)
	Peritumoral	0.700	0.727	0.667	0.727	0.667	0.713(0.611,0.838)
	Combined	0.825	0.818	0.833	0.857	0.789	0.846(0.753,0.939)
	Ensemble	0.813	0.773	0.861	0.872	0.756	0.865(0.782,0.948)
Test	Clinic	0.741	0.536	0.812	0.500	0.833	0.694(0.585,0.802)
	Intratumoral	0.704	0.679	0.712	0.452	0.864	0.701(0.589,0.813)
	Peritumoral	0.676	0.643	0.687	0.419	0.846	0.675(0.559,0.791)
	Combined	0.806	0.714	0.837	0.606	0.893	0.842(0.760,0.925)
	Ensemble	0.833	0.786	0.850	0.647	0.919	0.878(0.807,0.949)

**Table 4:** Statistical *p*-values from DeLong tests for pairwise comparisons of all models across the training, validation, and test cohorts.

Comparison Model	Clinic	Intratumoral	Peritumoral	Combined
Training				
Intratumoral	0.786			
Peritumoral	0.122	0.043		
Combined	0.007	<0.001	0.010	
Ensemble	<0.001	<0.001	<0.001	0.004
Validation				
Intratumoral	0.842			
Peritumoral	0.780	0.920		
Combined	0.079	0.018	0.012	
Ensemble	0.005	0.004	0.009	0.373
Test				
Intratumoral	0.921			
Peritumoral	0.820	0.758		
Combined	0.031	0.045	0.034	
Ensemble	<0.001	0.008	0.006	0.185

For the training cohort, the ensemble model exhibited an AUC of 0.925 (95% confidence interval [CI] 0.888–0.961), which was statistically significantly superior to the clinical model (p<0.001), intratumoral model (p<0.001), peritumoral model (p<0.001), and combined intratumoral-peritumoral model (p=0.004). In the validation cohort, the ensemble model achieved an AUC of 0.865 (95%CI 0.782–0.948), demonstrating significant superiority over the clinical model (p=0.005), intratumoral model (p=0.004), and peritumoral model (p=0.009). Regarding the test cohort, the ensemble model yielded an AUC of 0.878 (95%CI 0.807–0.949), which was significantly higher than the clinical model (p<0.001), intratumoral model (p=0.008), and peritumoral model (p=0.006). Additionally, the combined intratumoral-peritumoral model outperformed both the intratumoral and peritumoral models across all cohorts, with statistical significance.

**Figure 4:**
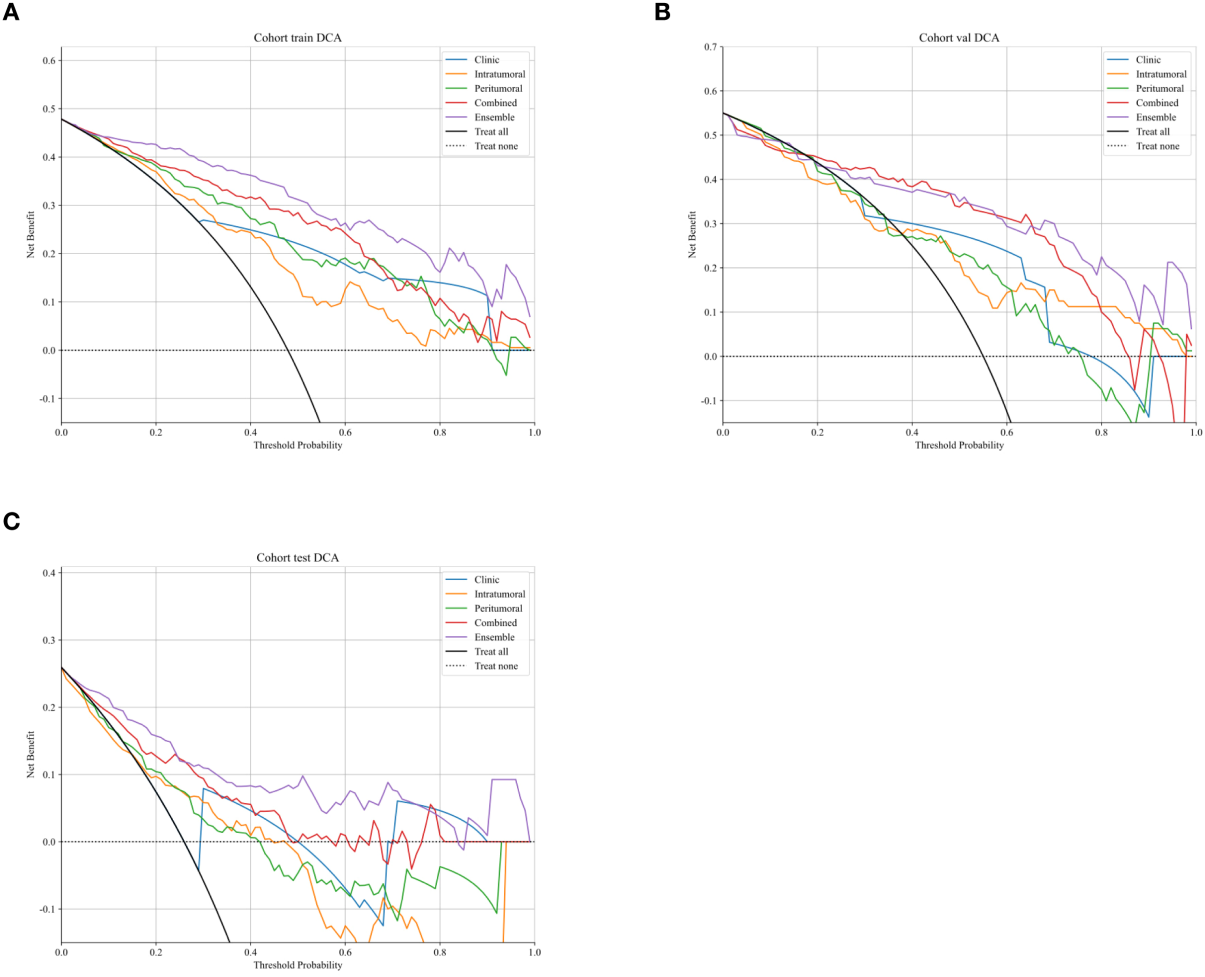
Decision curves analysis of different models for predicting TDs status in advanced gastric cancer in the training **(A)**, validation **(B)**, and test **(C)** cohorts.

**Figure 5:**
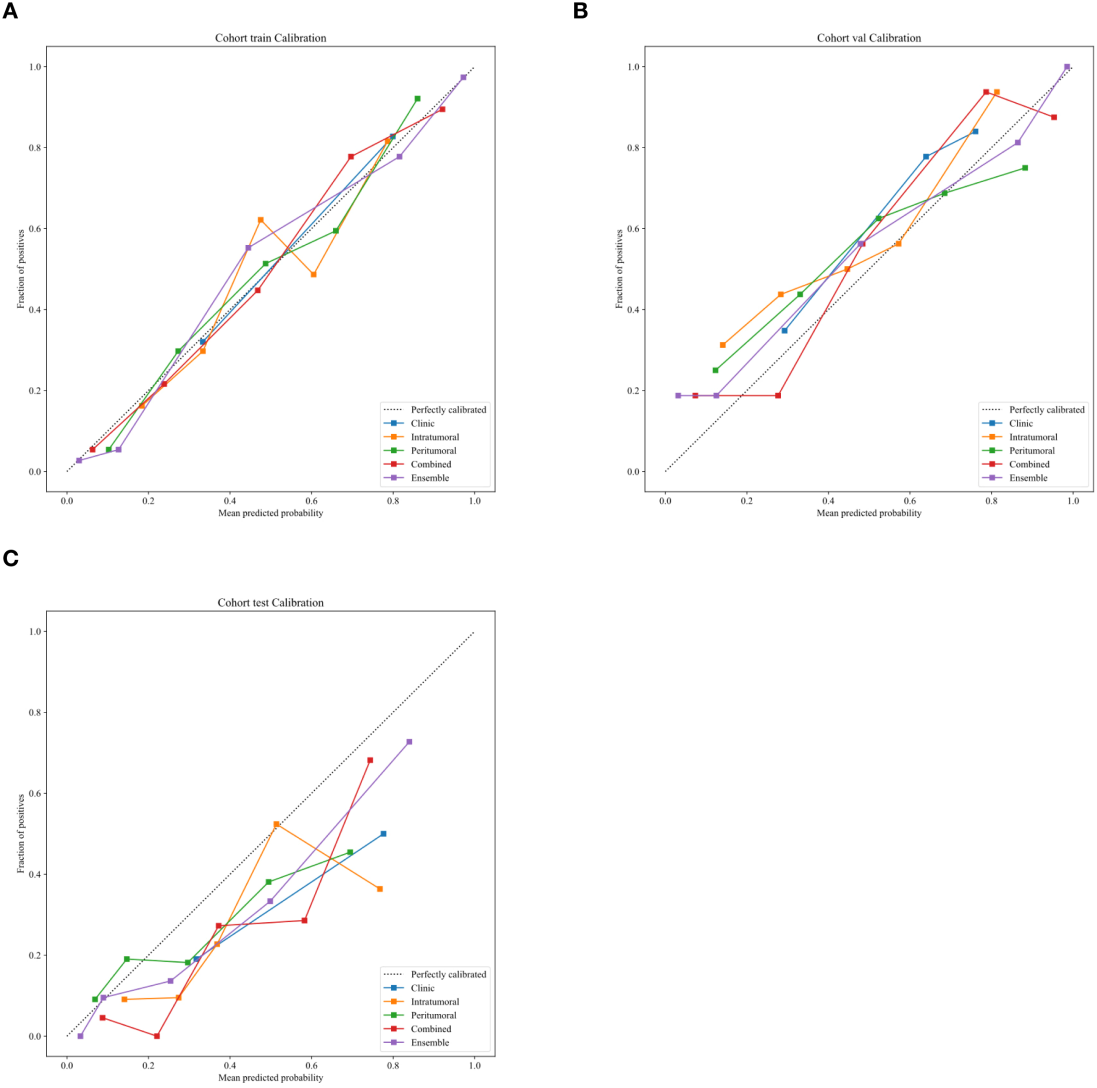
Calibration curves of different models for predicting TDs status in advanced gastric cancer in the training **(A)**, validation **(B)**, and test **(C)** cohorts.

**Figure 6:**
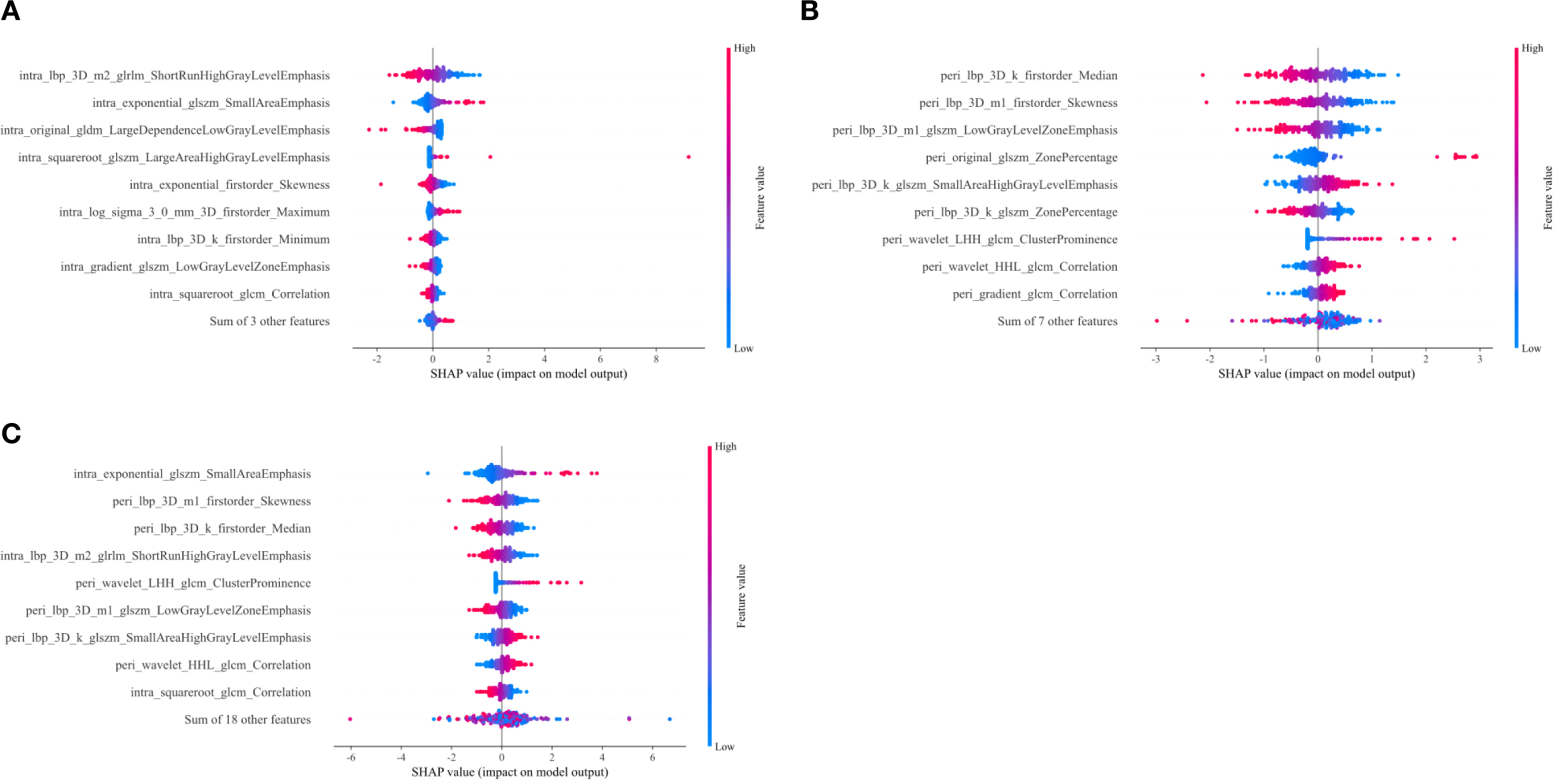
SHAP summary plots corresponding to the intratumoral **(A)**, peritumoral **(B)**, and combined intratumoral-peritumoral **(C)** radiomic models. These plots elucidate the model outputs by quantifying the impact of individual features on the predictive outcomes of each model. For each plot: the X-axis denotes SHAP values, which reflect the directional contribution of each feature to the prediction—positive values enhance the predicted probability of the target outcome (TDs positivity), while negative values reduce it; the Y-axis enumerates features ranked by their average SHAP importance (higher-ranked features exert greater predictive influence); each point corresponds to an individual observation, where color intensity denotes the feature value (blue represents low feature values, and red represents high feature values).SHAP, Shapley Additive exPlanations.

**Figure 7:**
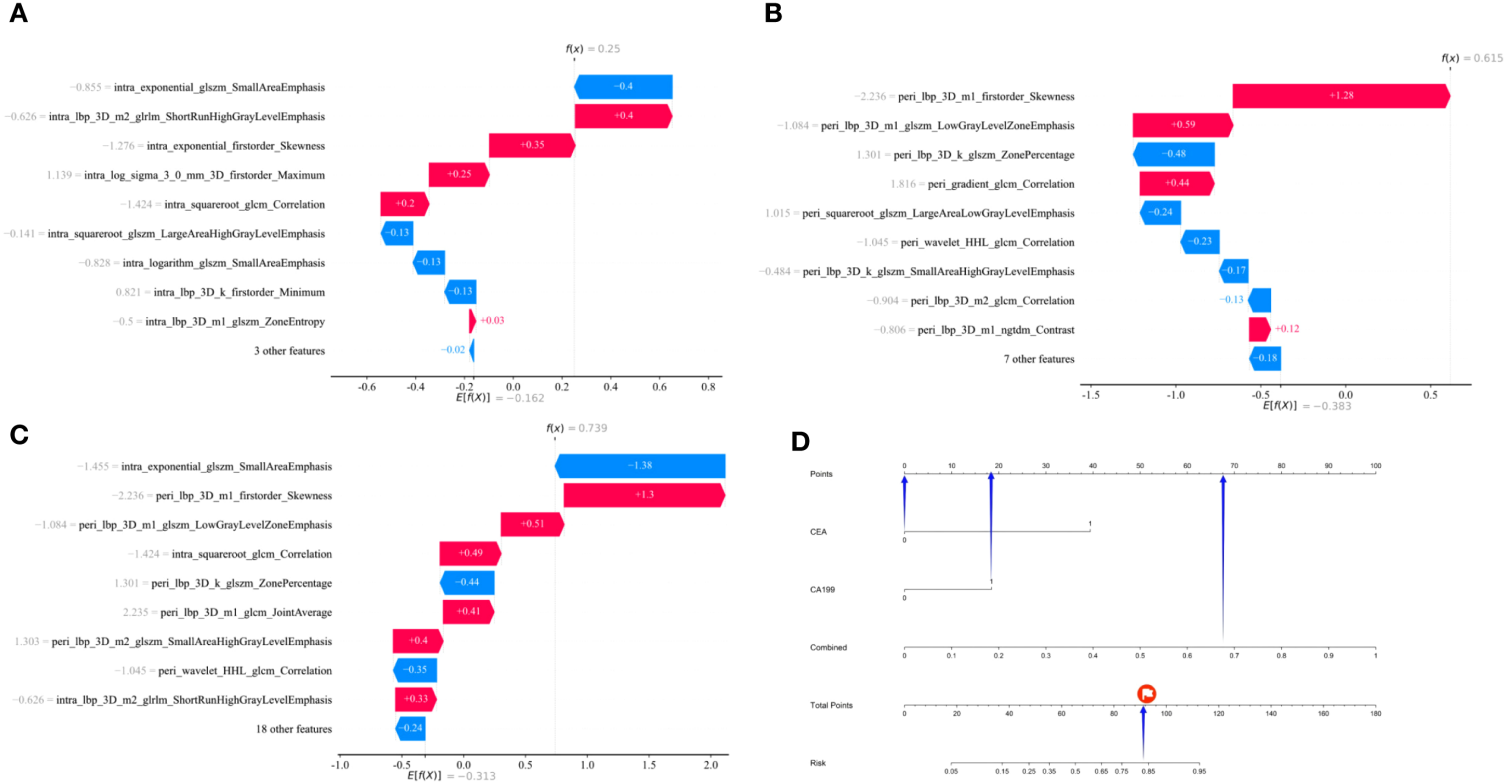
Model output interpretability analysis targeting a 56-year-old patient with positive TDs. SHAP waterfall plots corresponding to the intratumoral **(A)**, peritumoral **(B)**, and combined intratumoral-peritumoral **(C)** radiomic models for this patient. These plots elucidate the directional impact of individual features on the models’ predictive outcomes. For each plot: E[f(x)] denotes the baseline value, corresponding to the average model output across all samples; each bar represents the contribution of a single feature, where bar length indicates the magnitude of the impact, and color denotes the regulatory direction—red bars increase the prediction probability of TDs positivity relative to the baseline, while blue bars decrease it; f(x) refers to the final predictive outcome of the model (with higher f(x) values indicating a greater likelihood of TDs positivity). The predictive output of the ensemble model for this patient is illustrated via a nomogram **(D)**. “Combined” denotes the predicted probability derived from the combined intratumoral-peritumoral radiomic model. Using the point scale at the top of the nomogram, the points contributed by each feature (blue arrows) are determined. Summing these feature-specific points yields the total points (red marker), allowing mapping to the outcome at the bottom to obtain the final predicted probability of TDs positivity. SHAP, Shapley Additive exPlanations.

Decision curve analysis (DCA) revealed that the ensemble model yielded a higher net clinical benefit compared with the other models across various threshold probabilities (**Figure 4**). Calibration curve analysis demonstrated that the ensemble model exhibited superior predictive accuracy relative to both the individual radiomics models and the clinical model (**Figure 5**). SHAP (SHapley Additive exPlanations) analysis results for the constructed radiomics models are depicted in **Figure 6**. An illustrative example demonstrating the interpretability of the proposed radiomics model is presented in **Figure 7**.

## Discussion

In this study, we constructed and validated a series of radiomics models to investigate the potential of CT-derived radiomics features in providing predictive value for the preoperative assessment of TDs status in patients with AGC. Notably, the ensemble model—integrating the output of the combined intratumoral-peritumoral radiomics model with independent clinical prognostic factors—achieved the most robust performance. The reliability and generalizability of this ensemble model were fully corroborated by its consistently favorable performance across the training, validation and test cohorts.

Traditionally, the prognosis assessment of gastric cancer patients has primarily centered on pT stage, pN stage, and the absence of distant metastasis (25, 26). However, numerous studies have highlighted the inadequacy of the 8th edition AJCC TNM staging system in accurately stratifying outcomes for TDs-positive patients. For instance, Sun et al. (27) demonstrated that when stratified by pN category, significant differences in survival were observed between patients with and without TDs for those in pN0/pT1-3, pN1/pT3, pN2/pT1–3 and pN3/pT2–3 category. Mounting evidence has further confirmed that TDs represent an independent prognostic risk factor for GC: Zhou et al. (5) reported a 3-year disease-free survival (DFS) rate of 61.5% in the TDs-negative group, significantly higher than the 40.8% observed in the TDs-positive group (HR 1.78, 95% CI 1.44–2.21; P < 0.001). Beyond prognostic stratification, preoperative identification of TDs status also plays a pivotal role in optimizing surgical strategies and enhancing curative efficacy. Gong et al. (28) noted that TDs are frequently localized in the mesogastric region, a site that cannot be fully resected via conventional radical gastrectomy with D2 lymph node dissection. Consequently, D2 lymphadenectomy combined with complete mesogastric excision (D2+CME) is recommended for TDs-positive GC patients to achieve adequate tumor clearance. Based on the aforementioned findings, the precise preoperative diagnosis of TDs in GC patients holds profound clinical significance. Its value permeates key aspects including clinical decision-making optimization, individualized treatment formulation, accurate prognostic assessment, and improved reliability of tumor TNM staging—all of which exert a crucial impact on enhancing treatment outcomes and prolonging long-term survival for patients. Therefore, the development of an efficient and non-invasive method for preoperative TDs detection in GC patients addresses an urgent clinical unmet need and carries substantial practical value.

Radiomics has enabled the shift from “qualitative description” to “quantitative analysis” by quantifying latent features in gastric cancer imaging, demonstrating tremendous application value in preoperative diagnosis, precise staging, treatment response prediction, prognostic evaluation, and other aspects (16, 17). However, early studies mainly focused on intratumoral radiomics features (18, 29, 30). Notably, tumor invasion and metastasis are not isolated events but depend on the “remodeling” of the peritumoral microenvironment. Peritumoral radiomics features essentially represent quantitative imaging surrogates of pathophysiological changes in the tumor microenvironment (31, 32). Their biological significance lies in overcoming the limitation that intratumoral features only reflect the intrinsic characteristics of tumor cells, thereby more comprehensively revealing tumor invasiveness and the host’s antitumor response. Recent studies showed that integrating intratumoral and peritumoral radiomics features improves the performance of models in predicting tumor metastasis in colorectal cancer (CRC) and cervical cancer, with AUC values reaching approximately 0.80 in independent test cohorts (33, 34). Therefore, in our study, we incorporated both intratumoral and peritumoral radiomics features, and the predictive performance of the peritumoral radiomics model was not inferior to that of the intratumoral radiomics model. When the intratumoral and peritumoral radiomics features were combined, the predictive ability of the integrated model further improved, with an AUC of 0.878 in the training cohort, 0.838 in the validation cohort, and 0.853 in the test cohort. Thus, the additional value of peritumoral radiomics features in predicting the status of tumor deposits in gastric cancer was confirmed. It is important to note that this study adopted a 3D region of interest (3D ROI) segmentation strategy. Its core advantage lies in the comprehensive coverage of the entire tumor volume information, enabling more accurate quantification of tumor heterogeneity features (35). This approach effectively avoids the prevalent local sampling bias and subjective judgment bias associated with 2D ROI segmentation, thereby laying a solid methodological foundation for the development of high-performance radiomics prediction models.

Previous studies have shown that the presence of TDs is associated with multiple clinical and postoperative pathological features, which found that TDs are closely correlated with tumor diameter, Borrmann classification, tumor differentiation, pT, pN, pTNM, nerve invasion, and vascular invasion (12, 15). Given that the aim of our study was to develop a preoperative model for predicting TDs status in patients with gastric cancer, only clinical features were included in the analysis. Univariate and multivariate logistic regression analyses identified CEA and CA19–9 as independent predictive factors for TD status. Finally, an ensemble model was established by fusing the predictive outputs of the combined intratumoral-peritumoral radiomics model with the identified independent clinical predictors. Among all constructed models, the ensemble model achieved the highest area under the curve (AUC) values in the training (0.931, 95%CI 0.896-0.966), validation (0.880, 95%CI 0.807-0.983), and test cohorts (0.883, 95%CI 0.795-0.970).

Model interpretability is pivotal for the clinical translation of machine learning-based radiomics models. To address this critical requirement, the SHAP method was utilized to conduct interpretability analysis of the constructed radiomics models in the present study (36). Specifically, this approach quantifies the direction and magnitude of each feature’s contribution to individual patient-specific predictive outcomes, thereby facilitating comprehensive comprehension of the model’s decision-making mechanism among both clinicians and patients. Furthermore, a nomogram was developed to visualize the ensemble model, enabling clinicians to intuitively interpret predictive results and quantify the relative importance of each input variable. Collectively, the integration of these two complementary interpretability strategies significantly enhances the clinical applicability, trustworthiness, and acceptability of our predictive models.

Our study also has certain limitations. First, the retrospective design of this study may introduce inherent biases. Therefore, we plan to conduct multicenter prospective studies in the future to validate our findings. Second, our study used a fixed 2-mm peritumoral region for analysis, a predefined boundary that fails to fully reflect the biological heterogeneity of gastric cancer invasion and the anatomical adjacency of surrounding abdominal organs to the stomach. Future studies should optimize peritumoral delimitation by integrating radiological and pathological gold standards—for example, stratifying the peritumoral region by radial distances (2 mm, 5 mm, 10 mm) from the tumor margin and validating the optimal boundary with postoperative gastric cancer pathological sections. This optimized delimitation can exclude confounding from adjacent normal tissues and enhance the clinical relevance of extracted peritumoral radiomic features (37). Finally, we did not explore the correlation between the number of TDs and clinical or radiomic features. In future research, we intend to develop a preoperative predictive model for assessing the number of TDs, which is expected to provide more precise guidance for the individualized treatment of gastric cancer patients (38).

In conclusion, both intratumoral and peritumoral radiomic features can yield valuable insights into the status of TDs in patients with AGC. The ensemble model, integrating these two radiomic feature sets with clinical characteristics, exhibits superior performance and holds significant promise for guiding precision medicine in the management of AGC patients.

## Data Availability

The original contributions presented in the study are included in the article/**Supplementary Material**. Further inquiries can be directed to the corresponding author.
