# Supplementary material for: Preoperative prediction of tumor deposits in advanced gastric cancer using intratumoral and peritumoral CT radiomics: development and validation of an ensemble model
Source: Front Oncol. 2026 Mar 4;16:1763646. doi: 10.3389/fonc.2026.1763646 (PMC12995663; doi:10.3389/fonc.2026.1763646)
Supplement: Supplementary file 1 [file Table1.docx]

**Table S1.** Radiomic features used to build the model.

| Model | Radiomic features |
| --- | --- |
| Intratumoral model | intra_exponential_firstorder_Skewness |
|  | intra_exponential_glszm_SmallAreaEmphasis |
|  | intra_gradient_glszm_LowGrayLevelZoneEmphasis |
|  | intra_lbp_3D_k_firstorder_Minimum |
|  | intra_lbp_3D_m1_glszm_ZoneEntropy', |
|  | intra_lbp_3D_m2_glrlm_ShortRunHighGrayLevelEmphasis |
|  | intra_log_sigma_3_0_mm_3D_firstorder_Maximum |
|  | intra_logarithm_glszm_SmallAreaEmphasis |
|  | intra_original_gldm_LargeDependenceLowGrayLevelEmphasis |
|  | intra_squareroot_firstorder_Skewness |
|  | intra_squareroot_glcm_Correlation |
|  | intra_squareroot_glszm_LargeAreaHighGrayLevelEmphasis |
| Peritumoral model | peri_gradient_glcm_Correlation |
|  | peri_lbp_3D_k_firstorder_Median |
|  | peri_lbp_3D_k_firstorder_Skewness |
|  | peri_lbp_3D_k_glszm_SmallAreaHighGrayLevelEmphasis |
|  | peri_lbp_3D_k_glszm_ZonePercentage |
|  | peri_lbp_3D_m1_firstorder_Skewness |
|  | peri_lbp_3D_m1_glszm_LowGrayLevelZoneEmphasis |
|  | peri_lbp_3D_m1_ngtdm_Contrast |
|  | peri_lbp_3D_m2_glcm_Correlation |
|  | peri_log_sigma_2_0_mm_3D_glszm_LargeAreaLowGrayLevelEmphasis |
|  | peri_log_sigma_3_0_mm_3D_firstorder_Maximum |
|  | peri_original_glszm_ZonePercentage |
|  | peri_squareroot_glszm_LargeAreaLowGrayLevelEmphasis |
|  | peri_wavelet_HHL_glcm_Correlation |
|  | peri_wavelet_LHH_glcm_ClusterProminence |
|  | peri_wavelet_LLL_glszm_SizeZoneNonUniformity |
| Combined model | intra_exponential_firstorder_Skewness |
|  | intra_exponential_glszm_SmallAreaEmphasis |
|  | intra_gradient_glszm_LowGrayLevelZoneEmphasis |
|  | intra_lbp_3D_m1_glszm_ZoneEntropy |
|  | intra_lbp_3D_m2_glrlm_ShortRunHighGrayLevelEmphasis |
|  | intra_original_gldm_LargeDependenceLowGrayLevelEmphasis |
|  | intra_original_glszm_LargeAreaLowGrayLevelEmphasis |
|  | intra_square_gldm_LargeDependenceLowGrayLevelEmphasis |
|  | intra_squareroot_firstorder_Skewness |
|  | intra_squareroot_glcm_Correlation |
|  | intra_squareroot_glszm_LargeAreaHighGrayLevelEmphasis |
|  | peri_exponential_ngtdm_Coarseness |
|  | peri_lbp_3D_k_firstorder_Median |
|  | peri_lbp_3D_k_firstorder_Skewness |
|  | peri_lbp_3D_k_glszm_SmallAreaHighGrayLevelEmphasis |
|  | peri_lbp_3D_k_glszm_ZonePercentage |
|  | peri_lbp_3D_m1_firstorder_Skewness |
|  | peri_lbp_3D_m1_glcm_JointAverage |
|  | peri_lbp_3D_m1_glszm_LowGrayLevelZoneEmphasis |
|  | peri_lbp_3D_m2_glcm_Correlation |
|  | peri_lbp_3D_m2_glszm_SmallAreaHighGrayLevelEmphasis |
|  | peri_log_sigma_2_0_mm_3D_glszm_LargeAreaLowGrayLevelEmphasis |
|  | peri_square_glszm_LargeAreaEmphasis |
|  | peri_squareroot_glszm_LargeAreaLowGrayLevelEmphasis |
|  | peri_wavelet_HHL_glcm_Correlation |
|  | peri_wavelet_LHH_glcm_ClusterProminence |
|  | peri_wavelet_LLL_glszm_SizeZoneNonUniformity |
